# Tau phosphorylation regulates the interaction between BIN1’s SH3 domain and Tau’s proline-rich domain

**DOI:** 10.1186/s40478-015-0237-8

**Published:** 2015-09-23

**Authors:** Yoann Sottejeau, Alexis Bretteville, François-Xavier Cantrelle, Nicolas Malmanche, Florie Demiaute, Tiago Mendes, Charlotte Delay, Harmony Alves Dos Alves, Amandine Flaig, Peter Davies, Pierre Dourlen, Bart Dermaut, Jocelyn Laporte, Philippe Amouyel, Guy Lippens, Julien Chapuis, Isabelle Landrieu, Jean-Charles Lambert

**Affiliations:** INSERM, UMR 1167, Lille, France; Institut Pasteur de Lille, Lille, France; Université de Lille, Lille, France; CNRS, UMR 8576, Lille, France; FraBio IR 3688, Lille, France; Litwin-Zucker Research Center, Feinstein Institute for Medical Research, North-Shore Long Island Jewish Health System, Manhasset, NY USA; Department of Translational Medicine and Neurogenetics, IGBMC, INSERM U964, CNRS UMR7104, Université de Strasbourg, Illkirch, France; Center for Medical Genetics, Ghent University Hospital, Ghent, Belgium; Albert Einstein College of Medicine of Yeshiva University, New York, NY USA

## Abstract

**Introduction:**

The application of high-throughput genomic approaches has revealed 24 novel risk loci for Alzheimer’s disease (AD). We recently reported that the bridging integrator 1 (BIN1) risk gene is linked to Tau pathology.

**Results:**

We used glutathione S-transferase pull-down assays and nuclear magnetic resonance (NMR) experiments to demonstrate that BIN1 and Tau proteins interact directly and then map the interaction between BIN1’s SH3 domain and Tau’s proline-rich domain (PRD) . Our NMR data showed that Tau phosphorylation at Thr231 weakens the SH3-PRD interaction. Using primary neurons, we found that BIN1-Tau complexes partly co-localize with the actin cytoskeleton; however, these complexes were not observed with Thr231-phosphorylated Tau species.

**Conclusion:**

Our results show that (i) BIN1 and Tau bind through an SH3-PRD interaction and (ii) the interaction is downregulated by phosphorylation of Tau Thr231 (and potentially other residues). Our study sheds new light on regulation of the BIN1/Tau interaction and opens up new avenues for exploring its complex’s role in the pathogenesis of AD.

**Electronic supplementary material:**

The online version of this article (doi:10.1186/s40478-015-0237-8) contains supplementary material, which is available to authorized users.

## Introduction

Alzheimer’s disease (AD) is a progressive, neurodegenerative disorder characterized by (i) the massive loss of neurons in several regions of the brain. There are two distinct types of lesion: intraneuronal neurofibrillary tangles (NFTs, composed of abnormally phosphorylated Tau proteins) and extracellular amyloid deposits (composed of amyloid-β peptide (Aβ)). Mutations in the genes for amyloid precursor protein (APP), presenilin-1 and presenilin-2 are responsible for rare, autosomal-dominant forms of AD. The discovery of these mutations prompted the amyloid cascade hypothesis, which has radically changed our understanding of AD; APP metabolism and Aβ peptide production/degradation are thought to have a key role in the pathogenesis of AD (or at least the rare, familial forms of AD) [1]. However, the validity of this hypothesis in the vast majority of cases of AD (the so-called sporadic form) is subject to debate. Since 2009, the application of high-throughput genomic approaches has led to the characterization of 24 additional genetic risk factors for sporadic AD (following on from the apolipoprotein E (APOE) gene, which was characterized as a major genetic risk factor in 1993) [2]. We recently reported that the bridging integrator 1 (BIN1) gene is the first of these new genetic determinants for sporadic AD with a clear link to Tau pathology and (potentially) neurofibrillary degeneration [3]. We found that BIN1 was upregulated in the brains of AD cases and that a functional variant modulating BIN1 expression was associated with NFT loads. We were also able to show that BIN1 and Tau were directly interacting together *in vitro* and that human Tau toxicity observed in *Drosophila melanogaster* was partly suppressed by the silencing of the *Drosophila* BIN1 ortholog [3]. Taken as a whole, these data suggest that BIN1 interacts with Tau and is involved in Tau pathology. We therefore decided to characterize the BIN1-Tau interaction and the latter’s putative regulatory mechanisms in more detail. In the present work, we used glutathione S-transferase (GST) pull-down and nuclear magnetic resonance (NMR) experiments to show that BIN1’s SH3 domain interacts with Tau’s proline-rich domain (PRD). We found that amino acids [212–231] in Tau are essential for this interaction, and that phosphorylation within this sequence weakens the binding. Lastly, we used a proximity ligation assay (PLA) in primary neuron cultures to show that BIN1-Tau complexes co-localize with the actin cytoskeleton. However BIN1-Tau complexes were not observed in neurons when Tau Thr231 is phosphorylated. Taken as a whole, our results provide a detailed view of the molecular interplay between Tau and BIN1 and show for the first time that Tau phosphorylation weakens the interaction between these two proteins.

## Materials and methods

### cDNA and plasmids

Tau full-length (FL) 2N4R cDNA in pcDNA four was a kind gift from Luc Buée (INSERM U837, Lille, France). The BIN1 isoform used in the present study corresponds to the longest neuronal isoform 1 and will be denoted as BIN1 FL. For GST pull-down experiments, both Tau FL and Tau sub-fragment cDNA sequences were obtained by PCR with the primers described in Additional file 1, and subcloned into the pGEX-4 T2 vector (General Electric Healthcare Bio-Sciences, Piscataway, NJ, USA) to produce GST-Tau constructs. GST BIN1 FL and GST-BIN1/SH3 were obtained as previously described [4]. BIN1/ΔSH3 cDNA sequence was obtained by PCR from BIN1 FL cDNA (NM_139343.1) in PCMV6-XL5 (Origene, Rockville, MD, USA). For NMR experiments, the BIN1/SH3 domain cDNA was synthesized with optimized codons for recombinant expression in *E. coli* (Genecust, Dudelange, Luxembourg). The cDNA was subcloned between the *NdeI* and *XhoI* restriction sites in pET15b (Novagen, EMD Millipore, Darmstadt, Germany), thus allowing its expression with an N-terminal HisTag under the control of a T7 promoter. Recombinant Tau-F5[165-245] and TauFL were prepared for NMR experiments without a N-terminal tag with a pET15B vector. All cDNAs were checked by sequencing.

### Cell cultures and transfection

Human embryonic kidney 293 (HEK293) cells (CRL-1573 from LGC Standards/American Type Culture Collection, Molsheim, France) were cultured in Dulbecco’s modified Eagle’s medium (DMEM)/F12 (1:1) supplemented with 10 % fetal bovine serum, 2 mM glutamine, 20 units/ml penicillin and 20 μg/ml streptomycin (Gibco, LifeTechnologies, Carlsbad, CA, USA) in 5 % CO_2_ atmosphere and at 37 °C. Transient transfections were performed using Fugene-HD (Promega, Madison, WI, USA) according to the manufacturer’s instructions. Forty-eight hours later, cells were harvested in Tris-buffered saline (100 mM NaCl, 1 mM EDTA, 50 mM Tris–HCl) and centrifuged at 1000 g for 10 min at room temperature. Cell pellets were stored at−80 °C until processing for GST pull-down.

### The GST pull-down assay

The GST fusion proteins were expressed in *Escherichia coli* BL21(DE3) after induction with isopropyl 1-thio-β-D-galactopyranoside. Proteins were extracted from bacterial inclusion bodies by incubation with lysosyme for 1 h, overnight incubation with N-sarkosyl (0.001 %) and Triton X-100 (0.5 %), sonication and then centrifugation at 12,500 g for 30 min. All steps were performed at 4 °C. The GST fusion proteins were immobilized on glutathione-Sepharose beads (Pierce, ThermoFisher Scientific, Rockford, IL USA) according to the manufacturer’s instructions, and then incubated with HEK293 cell lysates for 1 h at room temperature. Beads were washed in Tris buffered saline, centrifuged at 10,500 g for 1 min and processed for SDS-PAGE analysis.

### Isotopic labelling and protein purification

Isotopic labelling of Tau and Tau-F5 was performed by growing recombinant BL21 (DE3) in minimal growth medium supplemented with ^15^N NH_4_Cl. The first purification step was performed by heating the bacterial protein extract for 15 min at 75 °C. The ^15^N Tau protein and ^15^N Tau[165–245] were recovered in the soluble fraction after centrifugation at 15,000 g for 30 min. The ^15^N Tau protein and ^15^N Tau-F5 were purified by cation exchange chromatography in 50 mM phosphate buffer pH 6.3, 1 mM EDTA (5 ml Hitrap SP Sepharose FF, General Electric Healthcare, Little Chalfont, United Kingdom). The pooled fractions from the chromatography purification step were transferred to ammonium bicarbonate by desalting on a 15/60 Hiprep desalting column (G25 resin, General Electric Healthcare) and lyophilized. The His-SH3 protein was purified on Ni-NTA resin, according to the manufacturer’s protocol.

### Acquisition and analysis of NMR spectra

1 mM d_4_-TMSP (3-(trimethylsilyl) propionate was used as an internal reference for proton chemical shifts (CSs) (0 ppm). The NMR buffer was 25 mM Tris-d11 pH 6.6, 30 mM NaCl, 2.5 mM EDTA and 1 mM DTT and 5 % D_2_O. Two-dimensional [^1^H, ^15^N] heteronuclear single quantum coherence (HSQC) spectra were recorded at 298 K on a Bruker 900 spectrometer equipped with a triple-resonance cryogenic probe (Bruker, Karlsruhe, Germany). Spectra were processed using Bruker TopSpin software (version 2.1, Bruker, Karlsruhe, Germany), and peaks were picked using Sparky software (version 3, T. D. Goddard and D. G. Kneller, University of California, San Francisco, CA, USA). The delta (δ) CSs of individual amide resonances of Tau-F5 and Tau FL were calculated with the following equation, while taking account of the relative dispersion of the proton and nitrogen CSs: δ(CS) = [((CS^1^H_bound_- CS^1^H_free_) + 0.2 (CS^15^N_bound_- CS^15^N_free_)) ^2^]^1/2^. The “bound” and “free” subscripts in the equation correspond to the CSs in the SH3-bound protein or the free protein, respectively.

### Phosphorylation of Tau protein

The CDK2/CycA3 protein was prepared and Tau was phosphorylated *in vitro* as previously described [5]. Enzymatic reactions were terminated by heating for 15 min at 75 °C and then centrifuged. The phosphorylation mixture was buffer-exchanged for NMR buffer using centrifugal desalting columns (Zeba Desalting Columns, with a 0.5 ml bed of G25 resin and a 7 kDa cut-off (Thermofisher Scientific, Waltham, MA USA)).

### Electrophoresis and Western blots

Samples were resuspended in Lithium Dodecyl Sulfate buffer supplemented with Nupage antioxidant, heated for 10 min at 95 °C, loaded and separated on a 4–12 % acrylamide gel (Nupage, Novex, Life Technologies, Carlsbad, CA, USA) and blotted on nitrocellulose membranes using a BioRad Trans-Blot transfer system kit (BioRad, Hercules, CA, USA) according to the manufacturer’s instructions. Membranes were blocked and probed with antibodies diluted at the concentration indicated in Additional file 2. All antibodies were purchased directly from the provider (described in Additional file 2), except for CP13 [6], RZ3 [7] and PHF1 [8]. Membranes were incubated with a horseradish-peroxidase-conjugated secondary antibody (Jackson Immunoresearch Laboratories, West Grove, PA, USA), and revealed by chemiluminescence (Luminata Classico^TM^, EMD Millipore) in a BioRad Chemidoc XRS system (BioRad). Immunoblot data were quantified with ImageLab software (BioRad). Coomassie staining was performed with 0.05 % Brilliant Blue G (mass/volume) in 50 % methanol (vol/vol) and 10 % acetic acid (v/v). Destaining was performed with a 25 % methanol (v/v) and 7 % acetic acid (v/v) solution.

### Immunofluorescence assays and PLAs

Cultured cells were fixed on glass coverslips with either 4 % paraformaldehyde (EMS, Hatfield, PA, USA) in Phosphate Buffered Saline (PBS) (Life Technologies). Fixed cells were washed and permeabilized for 10 min in PBS supplemented with 0.25 % Triton X-100, and then blocked in PBS supplemented with 2 % bovine serum albumin for 2 h at room temperature. Coverslips were incubated overnight at 4 °C with primary antibodies (as specified in Additional file 2), washed in PBS and incubated for 45 min with Alexa Fluor antibodies diluted at 1/400 from stock (Molecular Probes, Life Technologies). Alexa Fluor 647 phalloidin (Molecular Probes, Life Technologies)was used as per the manufacturer’s instructions to stain the actin cytoskeleton. For PLAs, the initial steps (from fixation to primary antibody incubation) were the same as those described above for immunofluorescence assays. The following steps (i.e., secondary antibody incubation, ligation, amplification and probing) were performed according to the manufacturer’s instructions (Olink Bioscience, Uppsala, Sweden). Images were acquired with a confocal microscope (LSM 710, Zeiss, Oberkochen, Germany) and processed using ZEN 2012 software (Zeiss). Three independent experiments were performed for each condition. The mean intensity per pixel in three different fields was measured using ImageJ software. Imaris software (Bitplane, Zurich, Switzerland) was used for three-dimensional (3D) image processing.

### Primary neuron cultures

Mixed cortical and hippocampal primary cultures were obtained from P0 rats, according to previously described procedures [9]. Briefly, hippocampi and cortices were isolated from newborn rats, and neurons were dissociated by trypsin digestion. Neurons were plated on poly-L-lysine-coated coverslips or six-well plates, and were incubated with Minimal Essential Medium (MEM) supplemented with 10 % fetal bovine serum, Glutamax, MEM vitamins and penicillin/streptomycin (Life Technologies), according to the manufacturer’s instructions. After 24 h, neurons were transferred into serum-free Neurobasal-A medium supplemented with B27 (Gibco, Life Technologies), Glutamax and uridine-deoxyfluorouridine for 14 days of in vitro culture. For Western blot analysis, primary neurons were directly harvested in LDS buffer supplemented with Nupage antioxidant (Life Technologies) and processed as described above in the “Electrophoresis and Western blots” section. For immunofluorescence assays, cells were fixed in 4 % paraformaldehyde (EMS) and processed as described above in the “Immunofluorescence assays and PLAs” section.

## Results

### BIN1’s SH3 domain interacts with Tau’s PRD

**Fig. 1:**
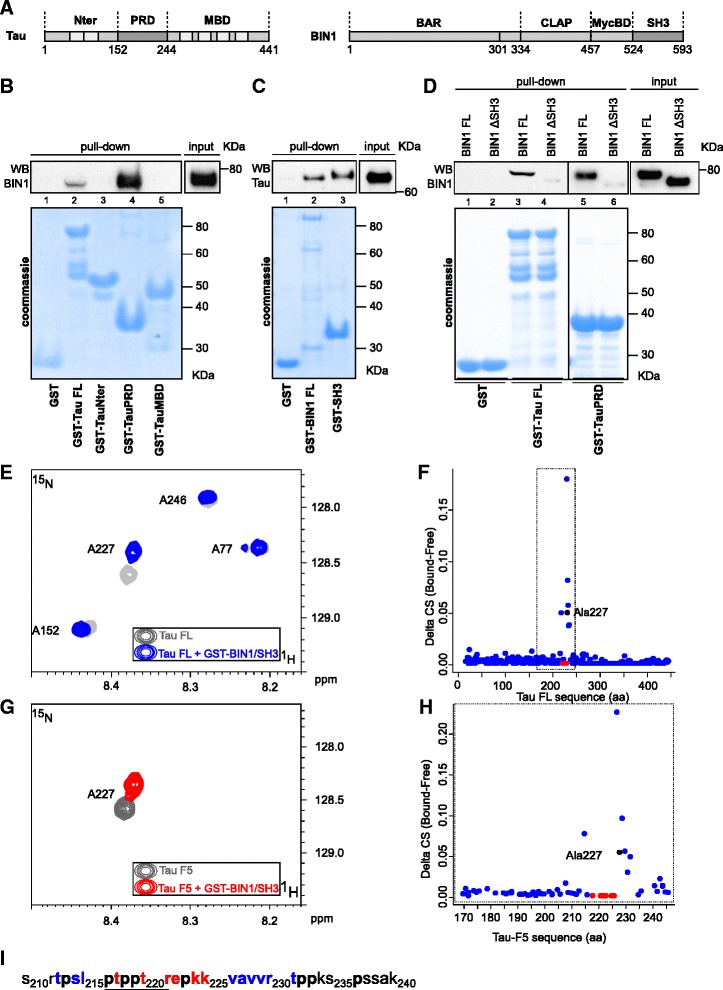
The BIN1-Tau interaction is mediated by the Tau PRD and the BIN1 SH3 domain. **a**. A schematic linear representation of 2N4R Tau (Tau FL) and BIN1 isoform1 (BIN1 FL) sequences and their respective domains (as used in GST pull-down assays). MBD: microtubule-binding domain; PRD: proline-rich domain; Bar: BIN1-amphiphysin-Rvs167; CLAP: clathrin and AP-2 binding; MycBD: Myc-binding domain; SH3: Src homology 3. **b**. GST pull-down assays performed with various Tau constructs corresponding to specific Tau subdomains (Lane 2: GST-Tau FL 2N4R; lane3: GST-Tau/Nter[1–152]; lane 4: GST-Tau/PRD[153–244]; lane 5: GST-Tau/MBD[244–441]) incubated with homogenates from BIN1-overexpressing HEK293 cells (*n* = 3). Upper panel: A representative immunoblot of BIN1 pull-down, revealed with a 99D anti-BIN1 antibody. **c**. GST pull-down assays performed either with full length BIN1 (Lane 2: GST-BIN1 FL) or the BIN1 SH3 domain (lane 3: GST-BIN1/SH3) incubated with homogenates from Tau FL-overexpressing HEK293 cells (*n* = 3). Upper panel: A representative immunoblot of Tau pull-down, revealed with a Dako anti-Tau antibody. **d**. GST pull-down assays performed with GST-Tau FL (lanes 3 and 4) or GST-Tau/PRD (lanes 5 and 6) incubated with homogenates from HEK293 cells overexpressing either BIN1 FL (lanes 3 and 5) or a BIN1 construct lacking the SH3 domain (BIN1/ΔSH3, lanes 4 and 6) (*n* = 3). Upper panel: A representative immunoblot of BIN1 pull-down, revealed with a 99D anti-BIN1 antibody. Lower panels (B, C, D): the corresponding Coomassie blue gels, used as loading controls for the pull-down assays. **e**. Two-dimensional (2D) [^1^H, ^15^N] HSQC spectra of 100 μM ^15^N 2N4R Tau, either free in solution (gray) or with a 1.6 molar amount of GST-BIN1/SH3 (blue, superimposed): Overlaid details of the full spectra presented in Additional file 3. **f**. Combined ^1^H, ^15^N CS perturbations (δ CS) in ppm, as defined in the Methods, in [^1^H, ^15^N] HSQC spectra of ^15^N 2N4R Tau with a 1.6 molar ratio of GST-BIN1/SH3 versus the molecule free in solution for every resonance along the sequence. The dashed box indicates the position of the Tau-F5 fragment within the Tau FL sequence. **g**. 2D [^1^H, ^15^N] HSQC spectra of 100 μM ^15^N Tau-F5 [165–245] free in solution (gray) or with a 1.2 molar amount of GST-BIN1/SH3 (red, superimposed): Overlaid details of full spectra presented in Additional file 4. **h**. Combined ^1^H, ^15^N CS perturbations (δ CS) in ppm, as defined in the Methods, in [^1^H, ^15^N] HSQC spectra of ^15^N Tau-F5 [165–245] with a 1.6 molar ratio of GST-BIN1/SH3, versus the free molecule in solution and for every resonance along the sequence. **i**. The *a minima* SH3 binding sequence in Tau. Proline residues are shown in bold, residues with resonance broadenings upon interaction are shown in red and residues with CS deviation when comparing free and bound states are shown in blue. The underlined amino acid residues fit the consensus sequence for SH3 binding (PxxPx+, where x is any residue and + is a positively charged residue) [30]

In previous work [3], we evidenced a direct interaction between full-length BIN1 and Tau *in vitro*. To further identify the protein domains involved in this interaction, we generated various constructs of Tau and BIN1 (Fig. 1a). We first incubated purified GST-Tau FL 1–441 or Tau domains (GST-Tau/N-terminal part (Nter), GST-Tau/PRD, GST-Tau/microtubule binding domain (MBD)) with HEK293 cell lysates overexpressing BIN1 FL. Only GST-Tau FL and GST-Tau/PRD pulled down BIN1 (Fig. 1b, lanes 2 and 4). In contrast, no interaction was observed with GST-Tau/Nter or (GST-Tau/MBD) (Fig. 1b, lanes 3 and 5). Secondly, we performed the reciprocal experiments by incubating Tau-overexpressing HEK293 cell lysates with either GST-BIN1 FL or GST-BIN1/SH3. In both cases, we observed an interaction with Tau FL (Fig. 1c). Lastly, we incubated GST-Tau FL or GST-Tau/PRD with HEK293 lysates overexpressing BIN1 FL or BIN1 lacking its SH3 domain. Removal of the BIN1 SH3 domain markedly weakened the BIN1-Tau interaction (Fig. 1d, lanes 4 and 6 vs lanes 3 and 5).

**Fig. 2:**
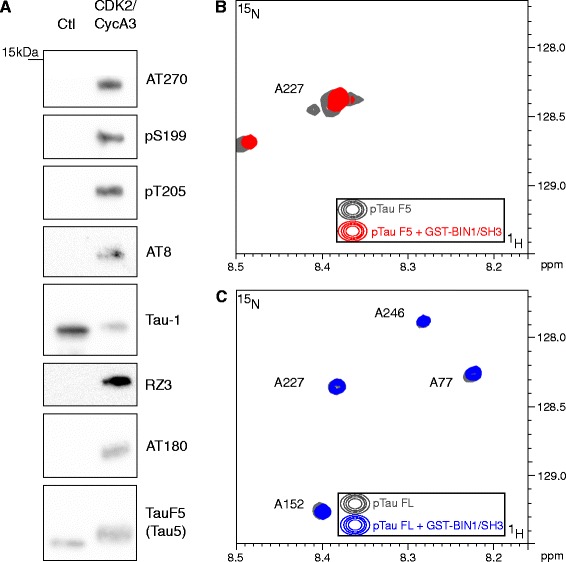
Tau phosphorylation precludes the BIN1-Tau interaction *in vitro*. **a**. Tau-F5 fragment phosphorylation analysis after *in vitro* phosphorylation by CDK2/CycA3 kinase. Representative immunoblots using various antibodies against phosphorylation epitopes in Tau. The total amount of Tau-F5 was revealed by the phosphorylation-independent antibody Tau5. The significant shift in molecular weight observed in CDK2/CycA3-treated samples indicates Tau hyperphosphorylation. In contrast to other antibodies, the Tau-1 antibody binds to various non-phosphorylated Tau sites; the signal thus decreases when Tau is hyperphosphorylated [31]. **b**. 2D [^1^H, ^15^N] HSQC spectra of 125 μM ^15^N CDK-phosphorylated Tau-F5[165–245] free in solution (gray) and with a 1 molar amount of GST-BIN1/SH3 (red, superimposed): Overlaid details of 2D [^1^H, ^15^N] HSQC spectra presented in Additional file 6. **c**. 2D [^1^H, ^15^N] HSQC spectra of 60 μM ^15^N CDK-phosphorylated 2N4R Tau free in solution (gray) and with a 2 molar amount of GST-BIN1/SH3 (blue, superimposed): Overlaid details of full spectra presented in Additional file 7

We next used NMR spectroscopy to determine which *a minima* sequence in Tau interacts with BIN1. Briefly, comparison of the ^1^H, ^15^N HSQC spectrum of Tau441 (which we and others have fully assigned [10]) with that of the protein in the presence of GST-BIN1/SH3 domain revealed perturbations in the CS of several amino acids (Fig. 1e, f, and Additional file 3). Analysis of the δ CS ([Tau + BIN1/SH3] *versus* [Tau]) for each Tau amino acid clearly delimited a short interaction region within the aa 212–231 sequence (Fig. 2f, i). These results were validated by studying a Tau domain encompassing aa 165 to 245 (Tau-F5) (Fig. 1g, h, and Additional file 4).

### In vitro, the BIN1-Tau interaction depends on Tau’s phosphorylation status

As Tau phosphorylation has been previously shown to regulate Tau PRD-SH3 interactions [11, 12], we next looked at whether Tau phosphorylation could interfere with the BIN1-Tau interaction. We acquired NMR data after the *in vitro* phosphorylation of Tau FL or a Tau fragment (Tau-F5[165–245]) by recombinant kinases (Fig. 2). Firstly, following *in vitro* incubation with CDK2/CycA3 [5], we confirmed that phosphorylation levels increased at several major Tau phosphoepitopes such as pT181 (AT270), pS199, pT205, pS202-T205 (AT8, Tau-1) and pT231 (RZ3, AT180), as shown by immunoblots (Fig. 2a) and NMR analysis (see Additional file 5). A comparison of NMR spectra of CDK2/CycA3-phosphorylated Tau-FL and Tau-F5 in the presence or absence of BIN1/SH3 did not reveal any significant CS perturbations. These findings indicated a lack of interaction between BIN1/SH3 domain and *in vitro*-phosphorylated Tau-F5 fragment or Tau-FL (Fig. 2b, c and Additional file 6, Additional file 7). Additional experiments with either a recombinant ERK kinase (see Additional file 8) or rat brain extract kinases (see Additional file 9) confirmed these findings. Taken as a whole, our results demonstrate that Tau phosphorylation in and around the PRD weakens the BIN1-Tau interaction by precluding SH3-PRD binding.

### BIN1-Tau complexes partly co-localized with the actin cytoskeleton network

**Fig. 3:**
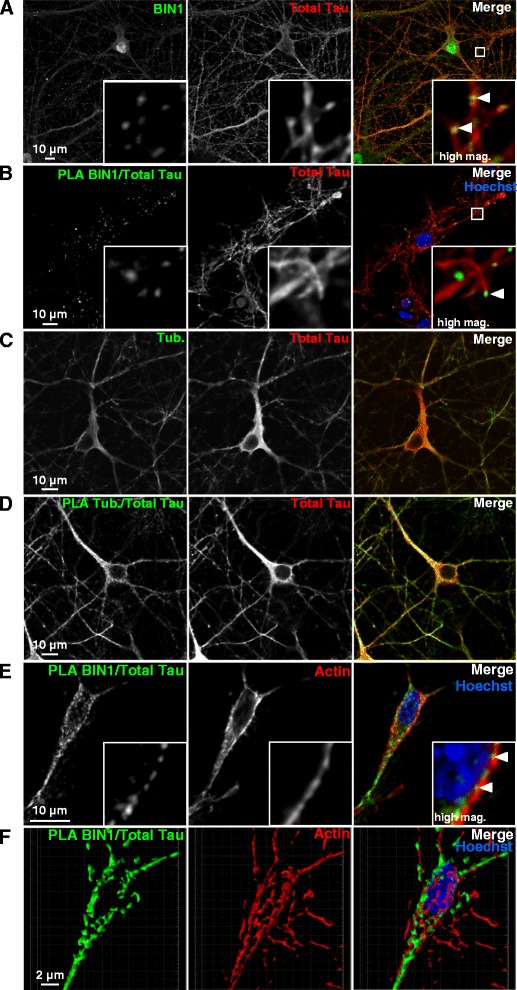
BIN1-Tau complexes partly co-localize with the actin cytoskeleton network. **a**. Immunofluorescence staining of endogenous BIN1 (green) and total Tau (red) in primary neuron cultures. Arrows indicate the co-localization of BIN1 and total Tau staining. **b**. PLAs (green) were been used to visualize endogenous BIN1-Tau complexes. Co-staining for total Tau (red) was compared with the PLA (BIN1/total Tau) signal. The arrow indicates the PLA signal located at the end of microtubule structures. **c**. Immunofluorescent staining of endogenous tubulin (tub, in green) and total Tau (red) in primary neuron cultures. **d**. A PLA (green) for visualizing endogenous complexes between tubulin and total Tau, combined with immunofluorescent staining of total Tau (red). **e**. A PLA for BIN1/total Tau (green) was combined with actin staining (red) using Alexa-Fluor633 phalloidin. The arrow shows the location of the PLA signal, with actin staining . **f**. A 3D image showing the proximity of the green (BIN1/total Tau) signal to the actin staining. A 3D video is presented in Additional file 11. Mag: magnification

**Fig. 4:**
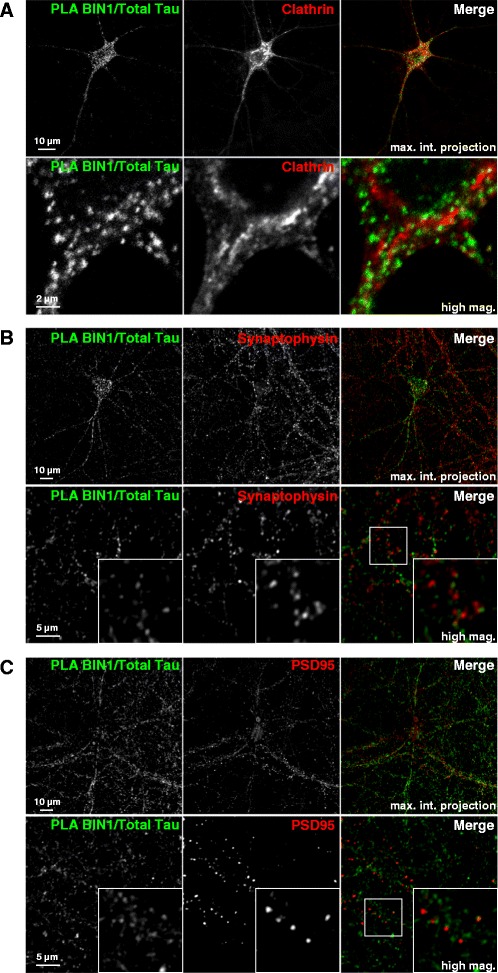
BIN1-Tau complexes are rarely co-localized with clathrin-coated membranes and are not co-localized with synaptic markers. **a**. A PLA for Tau-BIN1 (green) and clathrin staining (red) in primary neuron cultures. **b**. A PLA for Tau-BIN1 (green) and the pre-synaptic marker synaptophysin (red) in primary neuron cultures. **c**. A PLA for Tau-BIN1 (green) and the post-synaptic marker PSD95 (red) in primary neuron cultures. Upper panels: maximum intensity projection. Lower panels: a single confocal Z-stack. Mag: magnification

In order to further investigate the significance of BIN1-Tau binding in a physiological context, we used both conventional immunofluorescence experiments (Fig. 3a) and PLAs (Fig. 3b) to assess the intracellular location(s) of BIN1 and Tau in rat primary neurons. As described in the literature, Tau was found in the soma and the neurites (Fig. 3a-c), whereas BIN1 was found in the nucleus, the soma and the neurites (Fig. 3a). Within the neurites, we observed several puncta corresponding to the putative co-localization of BIN1-Tau (Fig. 3a). To validate these results, we used PLAs to detect potentially interacting BIN1 and Tau molecules (within 28 nm of each other). In agreement with the immunofluorescence experiments, a PLA signal for the BIN1 and Tau pair was identified in both the neuronal soma and the dendrites (Fig. 3b). This staining pattern contrasted with both the homogeneous immunofluorescence pattern observed for total Tau (Fig. 3c) and the strong PLA signal for Tau and tubulin (a major Tau partner) (Fig. 3d). Lastly, the BIN1-Tau PLA staining partly co-localized (mean ± standard deviation (SD) co-localization coefficient: 0.399 ± 0.041) with the actin cytoskeleton (as revealed by phalloidin staining; Fig. 3e, f, Additional file 10 and Additional file 11). In additional experiments, we further characterized the subcellular location of BIN1-Tau complexes in primary neurons (Fig. 4). Firstly, on the basis of the previously described role of BIN1 in endocytosis, we co-stained BIN1-Tau complexes and the endocytosis marker clathrin; the co-localization was very weak (Fig. 4a and Additional file 10; mean ± SD co-localization coefficient: 0.054 ± 0.014). Likewise, specific pre- or post-synaptic markers of functional neuronal compartments (such as synaptophysin (Fig. 4b) and PSD95 (Fig. 4c)) did not co-localize with BIN1-Tau complexes (mean co-localization coefficient ~0; see Additional file 10).

In conclusion, these results showed that Tau/BIN1 complexes are found at specific locations in primary neurons (as suggested by the dot-like staining) and partly co-localize with the actin cytoskeleton.

### Tau’s phosphorylation status influences BIN1-Tau binding in primary neurons

**Fig. 5:**
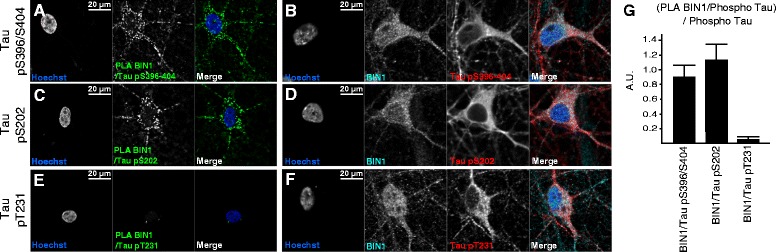
Specific Tau phosphorylation patterns for endogenous BIN1-Tau complexes in primary neuron cultures. A PLA (green) combining anti-BIN1 with either anti-phospho Tau pS396-404 **a**, pS202 **c**. or pT231 **e** antibodies. There was no PLA signal when anti-Tau pT231 was combined with anti-BIN1. Conventional immunofluorescence staining for BIN1 (cyan) and phospho-Tau (red) obtained with the antibodies used in the PLAs (pS396-404 **b**, pS202 **d** or pT231 **f**). **g**. Quantification of PLA fluorescence intensity in three fields and three independent experiments. The graph shows the ratio between the PLA intensity for BIN1/phosphoTau and phosphoTau alone. (*n* = 3) AU: arbitrary units. The error bars correspond to the SD

Since we had observed that Tau phosphorylation within or close to the Tau/PRD was able to modulate the BIN1-Tau interaction *in vitro*, we next looked at whether phosphorylation might modulate the interaction in primary neurons. To this end, we used a PLA that combined a BIN1 antibody with several specific, phosphorylation-dependent anti-Tau antibodies: an antibody against phosphorylation sites within Tau’s C-terminal region (pSer396-pSer404) (Fig. 5a, b) and two antibodies against phosphorylation sites near to or within the PRD peptide sequence that interacts with the BIN1/SH3 domain, i.e. pS202 (Fig. 5c, d) and pT231 (Fig. 5e, f). A PLA signal was observed with pSer202 and pSer396-pSer404 antibodies but not with antibodies against pThr231 (RZ-3, Fig. 5e, and AT180, data not shown). It should be noted that this difference cannot be attributed to differences in Tau phosphorylation levels in the various primary neuron cultures, since conventional immunofluorescence experiments did not highlight any obvious differences between the staining levels obtained for all the epitopes studied (Fig. 5b, d, f, g). These PLA results are in full agreement with those of our NMR experiments. Hence, in primary neurons, the phosphorylation status of the Tau/PRD sequence modulates interaction with the BIN1/SH3 domain. Moreover, our results suggest that Thr231 is one of the phosphorylation sites that modulates this interaction.

## Discussion

Progress in genetic analysis (notably in genome-wide association studies) has led to the identification of 24 validated risk loci for AD [13]. BIN1 is the first of these risk factors to have been linked to Tau pathology [3]. In the present study, we used biochemical and cellular approaches to further characterize the relationship between Tau and BIN1. Firstly, we found that BIN1’s SH3 domain interacts directly with the Tau PRD. Secondly, we determined that the amino acid sequence [212–231] within the PRD is involved in this interaction. Thirdly, we showed that Tau phosphorylation weakens the Tau-BIN1 interaction both *in vitro* and in primary neurons. Fourthly, we found that BIN1-Tau complexes exhibit a co-localization with the actin cytoskeleton in primary neurons.

BIN1 belongs to the amphiphysin protein family, the best-known function of which is to sense and generate membrane curvature through its BAR domain. This protein is also a key regulator of biological functions such as endocytosis, membrane recycling and cytoskeleton regulation [14]. Accordingly, BIN1 recruits some partners (such as AP-2 and clathrin) through its CLAP domain and others (such as dynamin and synaptojanin) through its SH3 domain [14]. Tau proteins are microtubule-associated proteins whose main function is to promote the polymerization and stabilization of the cytoskeleton microtubule network [15]. Tau is also involved in cell signaling pathways via the recruitment of kinases (such as Lck, Fyn, Src [16, 17] and phosphatidylinositol 3-kinase) and signaling adaptors (such as Grb2 [11, 18]). These interactions are mediated by Tau’s PRD. In the present work, we extended these results by showing that BIN1-Tau binding occurs through the SH3 domain and the PRD. Given that Tau phosphorylation (strong regulator of Tau function) is deregulated in AD, we sought to determine whether Tau phosphorylation was involved in regulation of the BIN1-Tau interaction. Our data indicate that Tau phosphorylation can indeed modulate the interaction between BIN1 and Tau *in vitro*. These results are consistent with previous reports of phosphorylation-dependent binding between Tau PRD and SH3 domains in proteins such as phosphatidylinositol 3-kinase, phospholipase C γ1, Grb2, and Src family kinases [11, 18]. Importantly, the results of our NMR and cell biology experiments suggest that phosphorylation at Thr231 (which is within the Tau PRD sequence that interacts with the BIN1 SH3 domain) weakens this interaction. These data are coherent with previous studies showing that Tau mutants mimicking phosphorylation at Thr231/Ser235 (T231D/S235D) bind 8 times less avidly (compared with native Tau) to the SH3 domain of Fyn [18], whereas a single mutation at Ser235 (S235E) does not affect the binding [11]. In contrast, mimicking phosphorylation at Ser396 and Ser404 (S396D/S404D) did not modify the affinity of 3-repeat Tau isoforms for Fyn/SH3. The latter literature data are consistent with (i) our PLA staining results for pS396-404 Tau and BIN1 and (ii) the fact that 3-repeat Tau isoforms are expressed in primary neuron cultures. Indeed, it has been well established that both 4-repeat and 3-repeat Tau isoforms are expressed in primary neurons after 14 days of in vitro culture [19]. Moreover, Tau proteins with different phosphorylation patterns (generated with various kinases) failed to interact with BIN1 *in vitro* (Fig. 3 and Additional file 8 and Additional file 9) but all were phosphorylated at Thr231. This strongly suggests that pThr231 is an important regulator of the BIN1/Tau interaction. Indeed, these findings were confirmed by our PLAs, since no interaction was observed with Thr231-phosphorylated Tau species in primary neurons. However, since Tau phosphorylation patterns are known to be associated with different subcellular locations (e.g. low Thr231 phosphorylation at the neuron’s cortical membrane [20]), one can argue that cell-sorting mechanisms might be responsible for the lack of interaction of BIN1 with pThr231 Tau in neurons. However, given that other sites in pThr231 Tau may also be phosphorylated, we cannot rule out the possibility that the latter also modify the BIN1/Tau interaction. Further experiments will be required to specify the possible contributions of other Tau phosphorylation sites and identify the mechanisms that underlie the regulation of the BIN1-Tau interaction by phosphorylation. Nevertheless, our finding that phosphorylation of Thr231 strongly influences the BIN1-Tau interaction highlights a putative link to the pathogenesis of AD. Indeed, Thr231 hyperphosphorylation occurs in the very early stages of neurofibrillary degeneration [21]. The enzymes that control this phosphorylation (such as Cdk5 [22], Gsk3-β [23], PP2A [24] and PIN1 [25]) are markedly deregulated during the AD process. These findings suggest that Tau hyperphosphorylation during AD (or at least phosphorylation at Thr231) can interfere with the physiological stoichiometry of BIN1-Tau complexes and may have a particular impact in the early stages of AD. We and others have shown that BIN1 expression is elevated in AD brains [3] and that BIN1 levels are correlated with the presence of neurofibrillary tangles [26]. Moreover, our previous work demonstrated that Tau toxicity in *Drosophila* models was suppressed by downregulating the *Drosophila* BIN1 ortholog [3]. Although our earlier results suggest that BIN1-Tau complexes have toxic effects, we cannot yet clearly define the roles of these complexes in AD or how Tau Thr231 phosphorylation contributes to this pathway during AD pathogenesis. In order to better understand the role of BIN1-Tau complexes, we performed experiments with markers of the various cell compartments involved in fundamental neuronal functions (such as synapses and endocytosis). Our results showed that BIN1-Tau complexes did not co-localize with either pre-synaptic or post-synaptic terminal markers. Moreover, clathrin (a marker of the endocytosis pathway) did not particularly co-localize with BIN1-Tau complexes. These results suggest that BIN1-Tau complexes exert their functions through cellular pathways other than the currently known pathogenic pathways involving Tau at the synapse [27] or involving BIN1 in endocytosis [14]. Nonetheless, we demonstrated substantial co-localization of BIN1-Tau complexes and the actin cytoskeleton. Therefore, one can postulate that BIN1 and microtubule-associated Tau have a role at the interface between actin and the microtubule network in neurons. Indeed, previous research has found that BIN1 interacts with the actin cytoskeleton [14] and serves as a membrane anchoring point for microtubules [28]. Further studies will be needed to fully understand the roles of BIN1-Tau complexes in neurons.

## Conclusions

Our results show that BIN1’s SH3 domain interacts directly with the Tau PRD, and that Tau phosphorylation (notably at Thr231) weakens this interaction. Taken as a whole, our findings highlight the link between BIN1, regulation of Tau phosphorylation and AD-related cellular pathways. Better knowledge of the interactions between Tau, BIN1 and the cytoskeleton in neurons may open up some exciting avenues for further research.

## Additional files

Additional file 1:
**Primer sequences used to amplify the constructs studied in the present work.** (PDF 37 kb) Additional file 2:
**Antibodies used and their respective dilutions. All dilutions refer to the stock solution provided by the manufacturer.** (PDF 55 kb) Additional file 3:
**BIN1-Tau interaction is mediated by the Tau sequence from aa 212 to aa 231.** 2D [^1^H, ^15^N] HSQC spectra of 100 μM ^15^N 2N4R Tau-FL free in solution (gray) or with a 1.6 molar amount of GST-BIN1/SH3 (blue, superimposed). The CS perturbations and peak broadening indicate the existence of an interaction. Annotated resonances correspond to the SH3 binding site, as defined in the sequence in Fig. 2e. (PDF 87 kb) Additional file 4:
**BIN1-Tau interaction is mediated by the Tau sequence from aa 212 to aa 231.** Overlaid detail of 2D [^1^H, ^15^N] HSQC spectra of 100 μM ^15^N Tau-F5 [165–245] free in solution (gray) or with a 1.2 molar amount of GST-BIN1/SH3 (red, superimposed). Blue arrows link the corresponding resonances in the free (gray) and bound (red) states. (PDF 74 kb) Additional file 5:
**Phosphorylation of Tau with the CDK2/CycA3 kinase.** A. Details of 2D [^1^H, ^15^N] HSQC spectra of 125 μM ^15^N Tau[165–245] (Tau-F5) phosphorylated with CDK2/CycA3 kinase. B. Details of 2D [^1^H, ^15^N] HSQC spectra of 100 μM ^15^N Tau phosphorylated with CDK2/CycA3 kinase. Shifted resonances corresponding to phosphorylated Ser and Thr residues are labelled [29]. Resonances located within the SH3 binding site are annotated in red. (PDF 76 kb) Additional file 6:
**Tau phosphorylation precludes the BIN1-Tau interaction in vitro.** 2D [^1^H, ^15^N] HSQC spectra of 125 μM ^15^N CDK-phosphorylated Tau-F5[165–245] free in solution (gray) and with a 1 molar amount of GST-BIN1/SH3 (red, superimposed). No CS perturbations or peak broadening were observed - indicating the absence of interaction between BIN1 and Tau-F5. (PDF 67 kb) Additional file 7:
**Tau phosphorylation precludes the BIN1-Tau interaction in vitro.** 2D [^1^H, ^15^N] HSQC spectra of 100 μM ^15^N CDK-phosphorylated 2N4R Tau, free in solution (gray) or with a 1.6 molar amount of GST-BIN1/SH3 (blue, superimposed). No CS perturbations or peak broadening were observed – indicating the absence of interaction between BIN1 and Tau Fl. (PDF 82 kb) Additional file 8:
**Interaction of GST-BIN1/SH3 with phospho-Tau.** A. In vitro phosphorylation of Tau by ERK kinase. Details of 2D [^1^H, ^15^N] HSQC spectra of ^15^N Tau-F5 [165–245] phosphorylated with ERK kinase. Shifted resonances corresponding to phosphorylated Ser and Thr residues are labelled [29]. Resonances of pT231 and pS235 are broader and less intense. Resonances located within the SH3 binding site are annotated in red. B. Interaction of GST BIN1/SH3 with phospho Tau-F5. HSQC spectra of 125 μM ^15^N ERK-phosphorylated Tau-F5 [165–245] free in solution (gray) and with a 1 molar amount of GST-BIN1/SH3(red, superimposed) C. Overlaid detail of 2D [^1^H, ^15^N] HSQC spectra presented in B. For details of the methods, see Additional file 12. (PDF 123 kb) Additional file 9:
**Interaction of GST-BIN1/SH3 with phospho-Tau.** A. in vitro phosphorylation of Tau by rat brain extracts. Details of 2D [^1^H, ^15^N] HSQC spectra of ^15^N Tau phosphorylated with rat brain extracts. Shifted resonances corresponding to phosphorylated Ser and Thr residues are labelled [29]. Resonances of pT231 and pS235 are broader and less intense. Resonances located within the SH3 binding site are annotated in red. B. Interaction of phosphorylated Tau FL with GST-BIN1/SH3. 2D [^1^H, ^15^N] HSQC spectra of 100 μM ^15^N Tau phosphorylated with rat brain extract, free in solution (gray) or with a 1.6 molar amount of GST-BIN1/SH3 (blue, superimposed). C. Overlaid detail of 2D [^1^H, ^15^N] HSQC spectra presented in B. For details of the methods, see Additional file 12. (PDF 137 kb) Additional file 10:
**Co-localization measurement of BIN1-Tau complexes with actin, clathrin-coated membranes and synapse terminal markers.** PLA Tau-BIN1 staining (green) with (A) actin staining, (B) clathrin staining, (C) the pre-synaptic marker synaptophysin and (D) the post-synaptic marker PSD95 (red) in primary neuron cultures. The right-hand panels show the pixels with co-localization (in white) of PLA and the various markers. The co-localization coefficient was calculated according to Mander’s method using ZEN 2012 software. The red channel was used as a reference. E. A graph showing the mean ± SD (error bar) coefficient for co-localization between PLA BIN1/Tau and the indicated markers (*n* = 6). For details of the methods, see Additional file 12. (PDF 3475 kb) Additional file 11:
**A movie showing 3D images processed with Imaris software.** The PLA (BIN1/total Tau) signal (green) was located in the vicinity of the actin staining (red) at the membrane. (ZIP 10MB) Additional file 12:
**Supplementary methods.** (PDF 89 kb)
