# Supplementary figures and images for: Tau phosphorylation regulates the interaction between BIN1’s SH3 domain and Tau’s proline-rich domain
Source: Acta Neuropathol Commun. 2015 Sep 23;3:58. doi: 10.1186/s40478-015-0237-8 (PMC4580349; doi:10.1186/s40478-015-0237-8)

Additional File 9

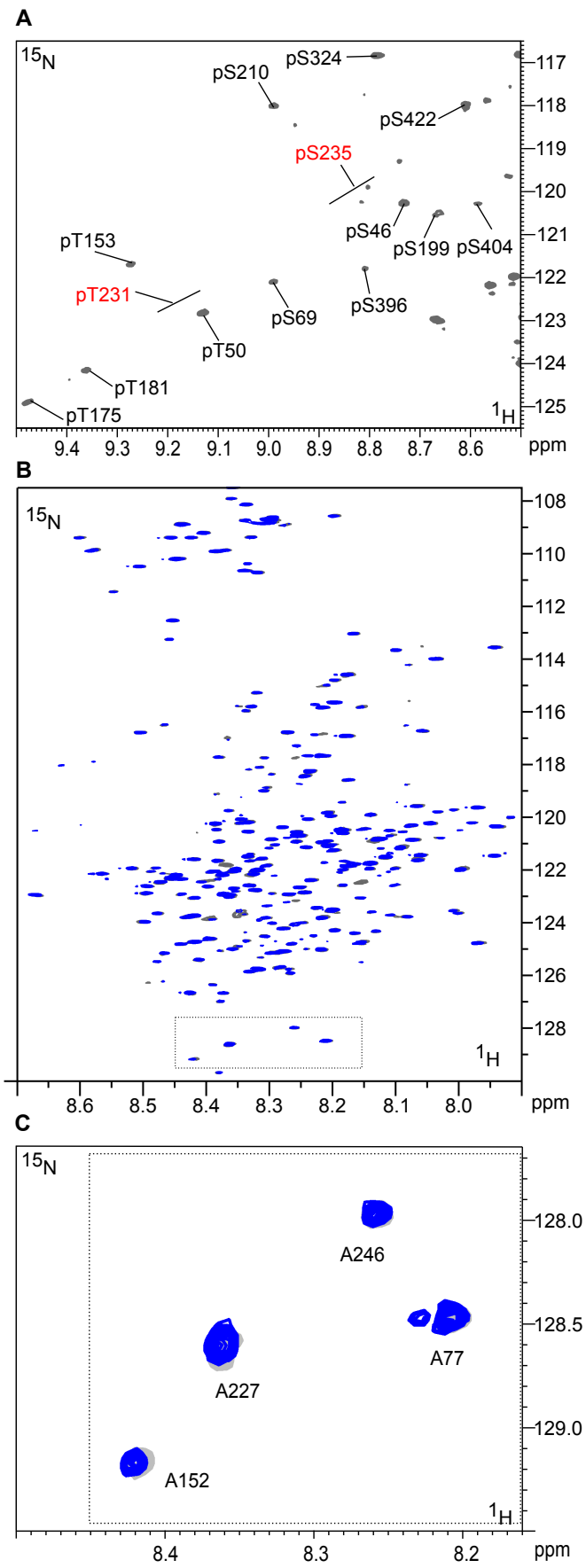

Supplement: Additional file 1: — Primer sequences used to amplify the constructs studied in the present work. (PDF 37 kb) [file 40478_2015_237_MOESM9_ESM.pdf]

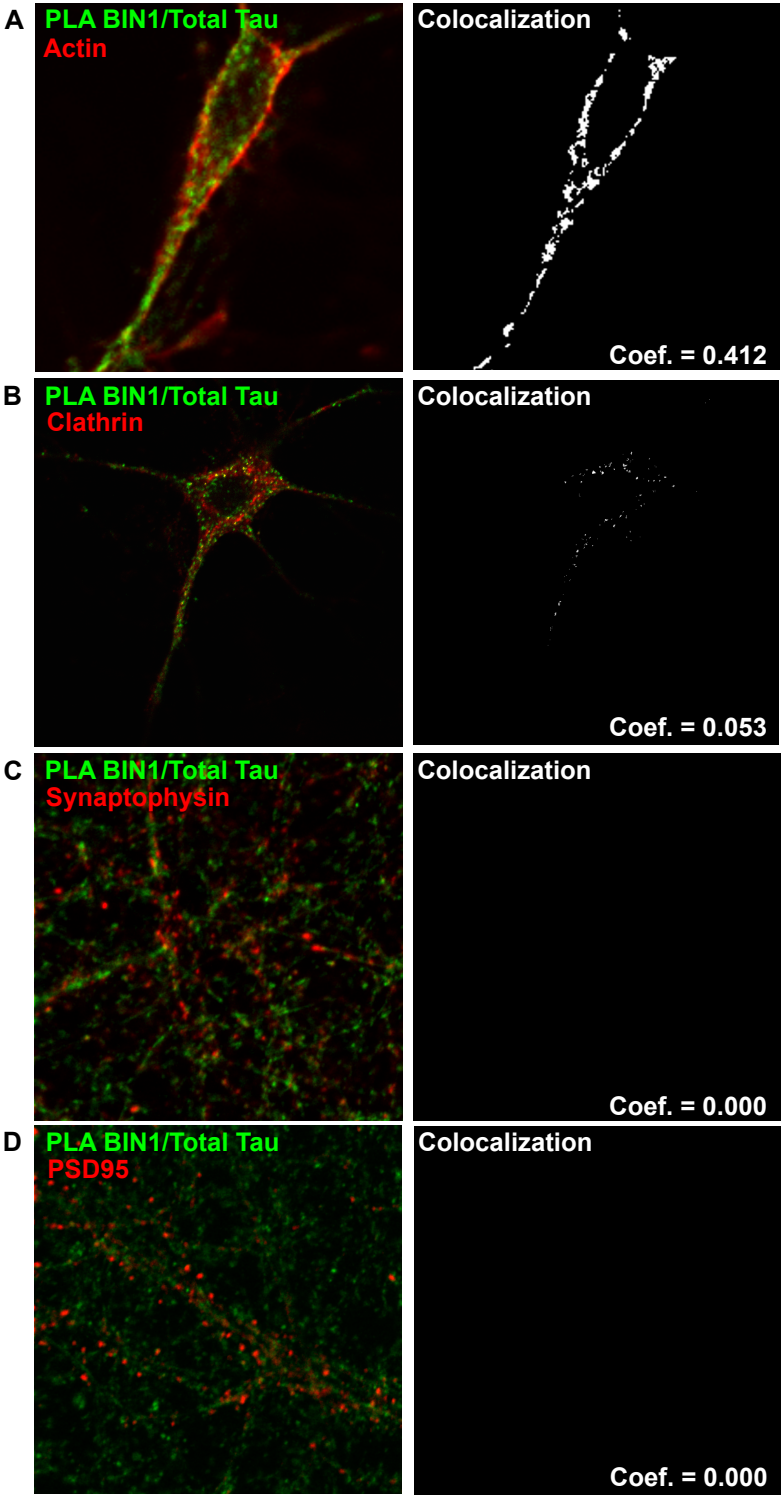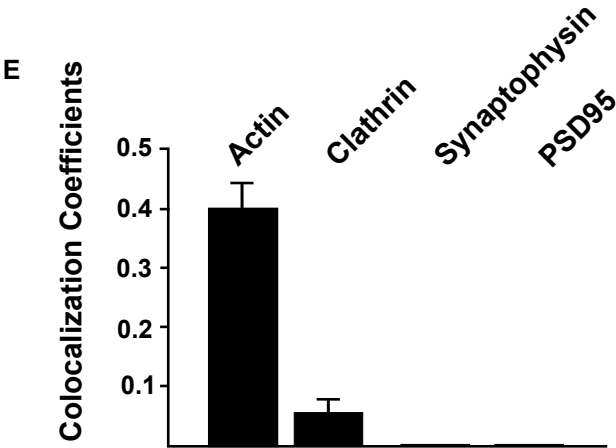

Supplement: Additional file 2: — Antibodies used and their respective dilutions. All dilutions refer to the stock solution provided by the manufacturer. (PDF 55 kb) [file 40478_2015_237_MOESM10_ESM.pdf]

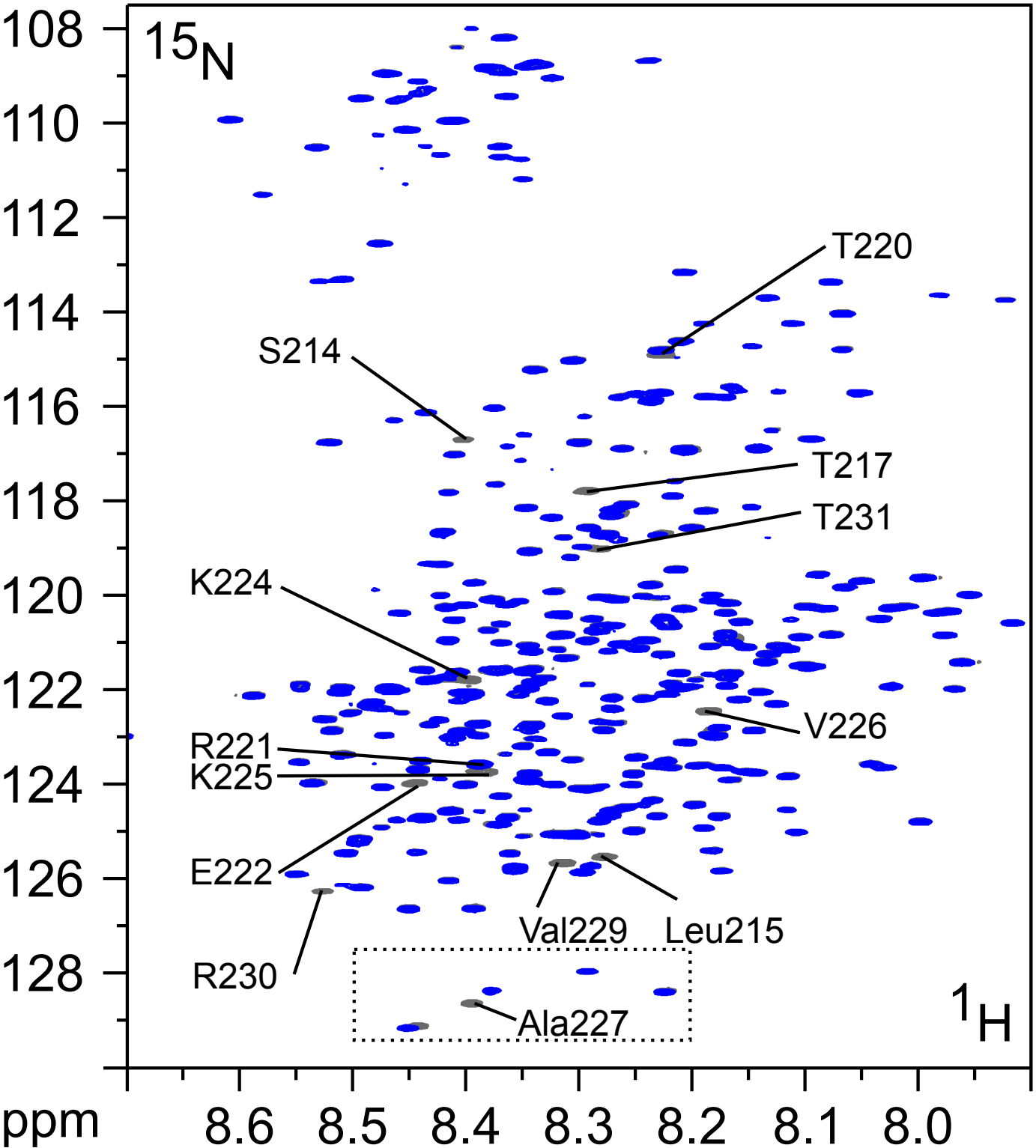

Supplement: Additional file 5: — Phosphorylation of Tau with the CDK2/CycA3 kinase. A. Details of 2D [1H, 15N] HSQC spectra of 125 μM 15N Tau[165–245] (Tau-F5) phosphorylated with CDK2/CycA3 kinase. B. Details of 2D [1H, 15N] HSQC spectra of 100 μM 15N Tau phosphorylated with CDK2/CycA3 kinase. Shifted resonances corresponding to phosphorylated Ser and Thr residues are labelled [29]. Resonances located within the SH3 binding site are annotated in red. (PDF 76 kb) [file 40478_2015_237_MOESM3_ESM.pdf]

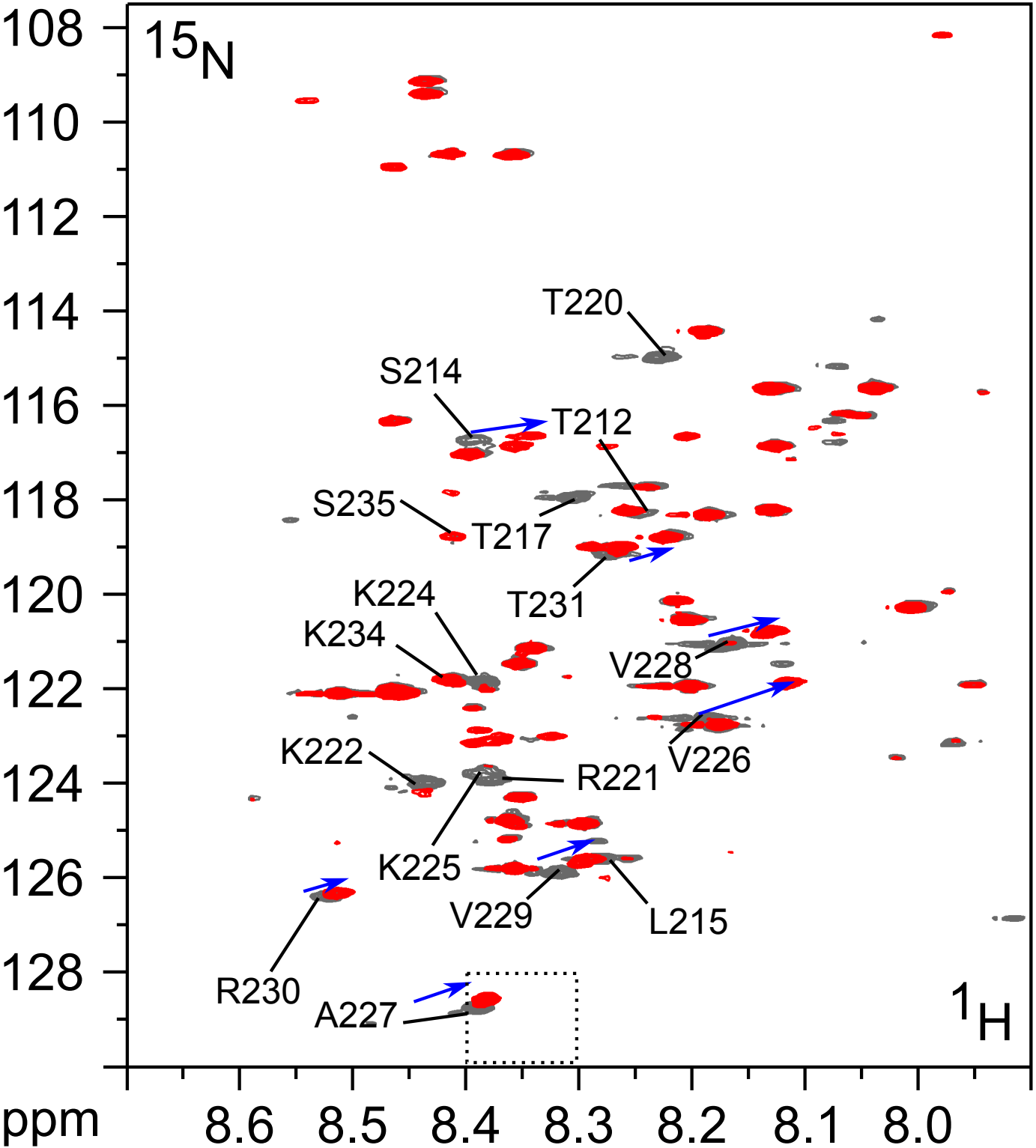

Supplement: Additional file 6: — Tau phosphorylation precludes the BIN1-Tau interaction in vitro. 2D [1H, 15N] HSQC spectra of 125 μM 15N CDK-phosphorylated Tau-F5[165–245] free in solution (gray) and with a 1 molar amount of GST-BIN1/SH3 (red, superimposed). No CS perturbations or peak broadening were observed - indicating the absence of interaction between BIN1 and Tau-F5. (PDF 67 kb) [file 40478_2015_237_MOESM4_ESM.pdf]

Additional File 5

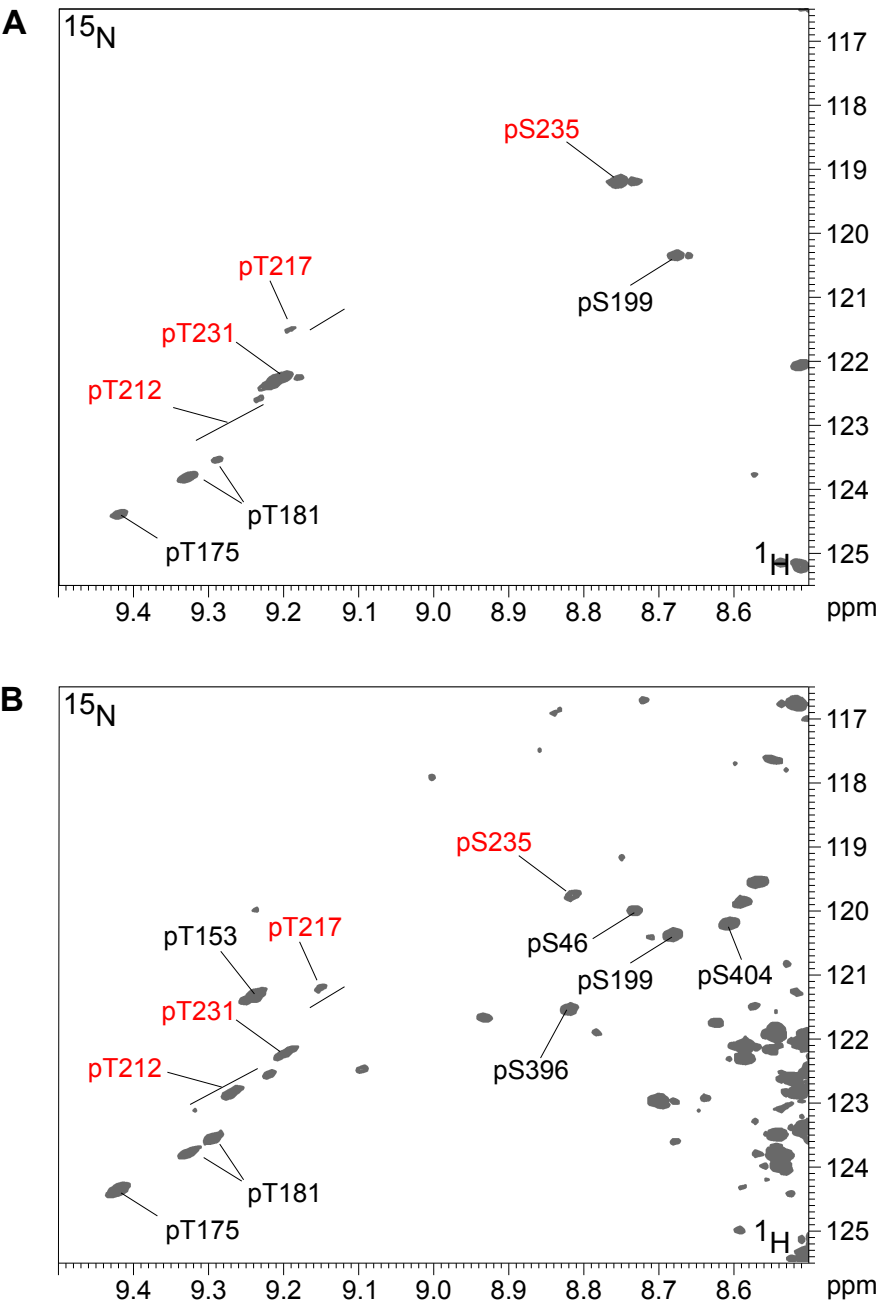

Supplement: Additional file 7: — Tau phosphorylation precludes the BIN1-Tau interaction in vitro. 2D [1H, 15N] HSQC spectra of 100 μM 15N CDK-phosphorylated 2N4R Tau, free in solution (gray) or with a 1.6 molar amount of GST-BIN1/SH3 (blue, superimposed). No CS perturbations or peak broadening were observed – indicating the absence of interaction between BIN1 and Tau Fl. (PDF 82 kb) [file 40478_2015_237_MOESM5_ESM.pdf]

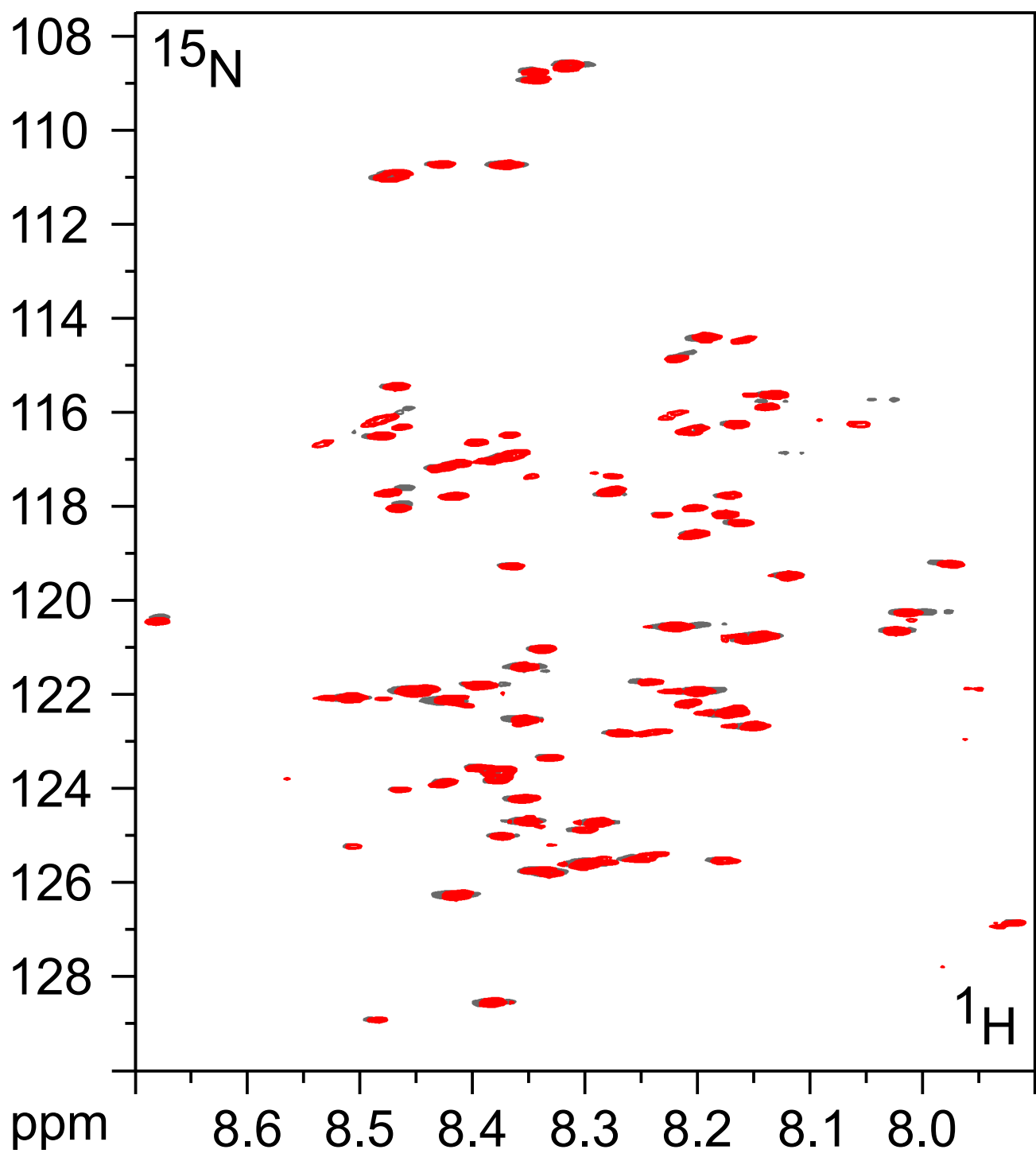

Supplement: Additional file 8: — Interaction of GST-BIN1/SH3 with phospho-Tau. A. In vitro phosphorylation of Tau by ERK kinase. Details of 2D [1H, 15N] HSQC spectra of 15N Tau-F5 [165–245] phosphorylated with ERK kinase. Shifted resonances corresponding to phosphorylated Ser and Thr residues are labelled [29]. Resonances of pT231 and pS235 are broader and less intense. Resonances located within the SH3 binding site are annotated in red. B. Interaction of GST BIN1/SH3 with phospho Tau-F5. HSQC spectra of 125 μM 15N ERK-phosphorylated Tau-F5 [165–245] free in solution (gray) and with a 1 molar amount of GST-BIN1/SH3(red, superimposed) C. Overlaid detail of 2D [1H, 15N] HSQC spectra presented in B. For details of the methods, see Additional file 12. (PDF 123 kb) [file 40478_2015_237_MOESM6_ESM.pdf]

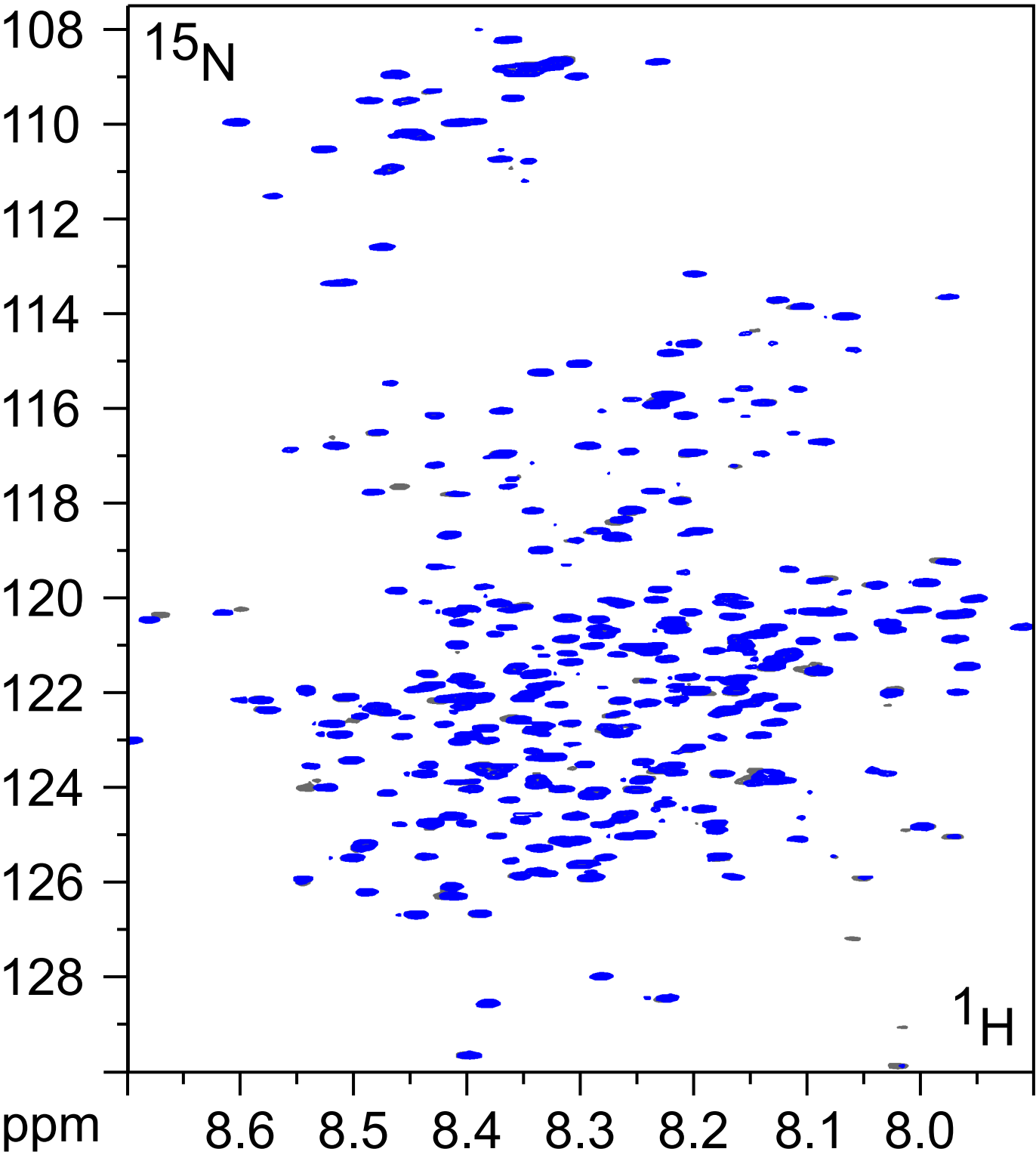

Supplement: Additional file 9: — Interaction of GST-BIN1/SH3 with phospho-Tau. A. in vitro phosphorylation of Tau by rat brain extracts. Details of 2D [1H, 15N] HSQC spectra of 15N Tau phosphorylated with rat brain extracts. Shifted resonances corresponding to phosphorylated Ser and Thr residues are labelled [29]. Resonances of pT231 and pS235 are broader and less intense. Resonances located within the SH3 binding site are annotated in red. B. Interaction of phosphorylated Tau FL with GST-BIN1/SH3. 2D [1H, 15N] HSQC spectra of 100 μM 15N Tau phosphorylated with rat brain extract, free in solution (gray) or with a 1.6 molar amount of GST-BIN1/SH3 (blue, superimposed). C. Overlaid detail of 2D [1H, 15N] HSQC spectra presented in B. For details of the methods, see Additional file 12. (PDF 137 kb) [file 40478_2015_237_MOESM7_ESM.pdf]

Additional File 8

**A**

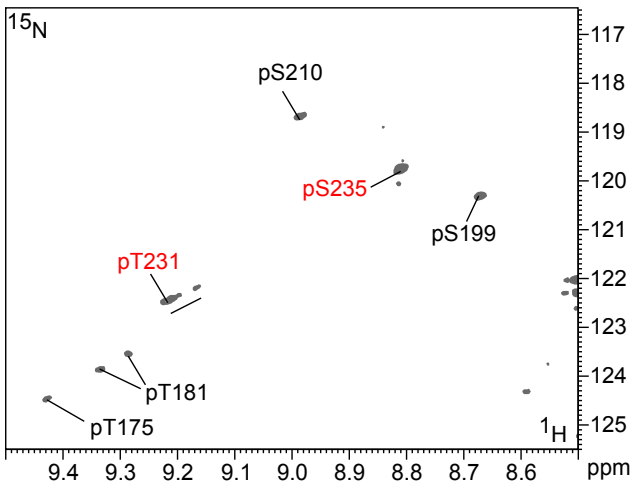

**B**

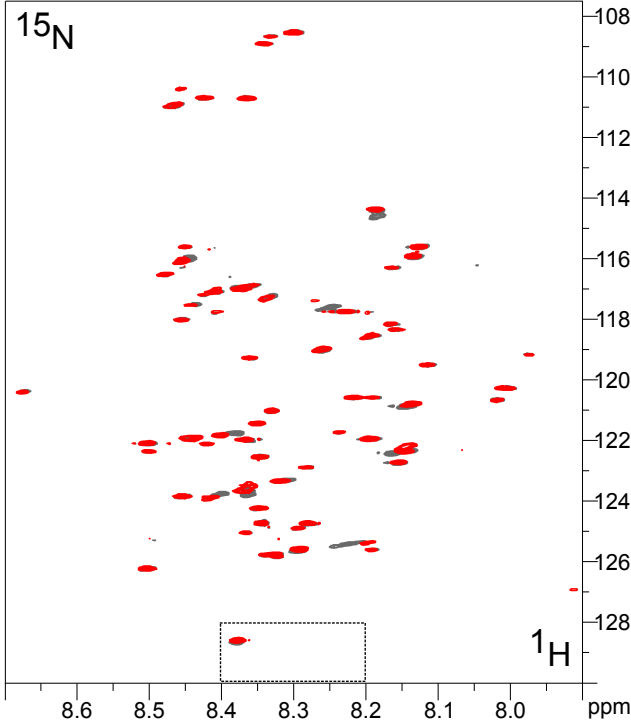

**C**

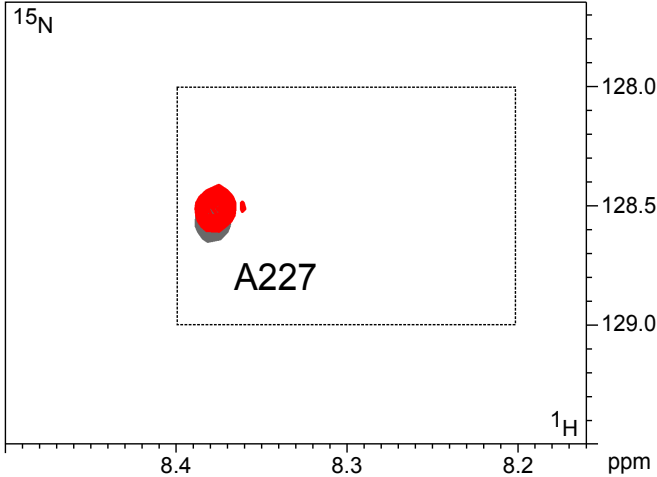

Supplement: Additional file 10: — Co-localization measurement of BIN1-Tau complexes with actin, clathrin-coated membranes and synapse terminal markers. PLA Tau-BIN1 staining (green) with (A) actin staining, (B) clathrin staining, (C) the pre-synaptic marker synaptophysin and (D) the post-synaptic marker PSD95 (red) in primary neuron cultures. The right-hand panels show the pixels with co-localization (in white) of PLA and the various markers. The co-localization coefficient was calculated according to Mander’s method using ZEN 2012 software. The red channel was used as a reference. E. A graph showing the mean ± SD (error bar) coefficient for co-localization between PLA BIN1/Tau and the indicated markers (n = 6). For details of the methods, see Additional file 12. (PDF 3475 kb) [file 40478_2015_237_MOESM8_ESM.pdf]
