# Supplementary material for: Tau phosphorylation regulates the interaction between BIN1’s SH3 domain and Tau’s proline-rich domain
Source: Acta Neuropathol Commun. 2015 Sep 23;3:58. doi: 10.1186/s40478-015-0237-8 (PMC4580349; doi:10.1186/s40478-015-0237-8)
Supplement: Additional file 3: — BIN1-Tau interaction is mediated by the Tau sequence from aa 212 to aa 231. 2D [1H, 15N] HSQC spectra of 100 μM 15N 2N4R Tau-FL free in solution (gray) or with a 1.6 molar amount of GST-BIN1/SH3 (blue, superimposed). The CS perturbations and peak broadening indicate the existence of an interaction. Annotated resonances correspond to the SH3 binding site, as defined in the sequence in Fig. 2e. (PDF 87 kb) [file 40478_2015_237_MOESM1_ESM.pdf]

Primer sequences used to amplify the various constructs used

Tau FL	ggatcc-ATGGCTGAGCCCCGCCAGGA **BamHI**	ctcgag-TCACAAACCCTGCTTGGCCA **XhoI**
Tau Nter	ggatcc-ATGGCTGAGCCCCGCCAGGA **BamHI**	ctcgag-TCAGGCGATCTTCGTTTTACCAT **XhoI**
Tau PRD	ggatcc-ACACCGCGGGGAGCAGCCCC **BamHI**	ctcgag-TCACAGGCGGCTCTTGGCGGAAG **XhoI**
Tau MBD	ggatcc-CAGACAGCCCCCGTGCCCAT **BamHI**	ctcgag-TCACAAACCCTGCTTGGCCA **XhoI**
BIN1deltaSH3	GGCTGGGAGCGCGGCGCGC	aagctt-TCATGGGGGCAGGTCCAAGCGCC **HindIII**
