# Supplementary material for: Tau phosphorylation regulates the interaction between BIN1’s SH3 domain and Tau’s proline-rich domain
Source: Acta Neuropathol Commun. 2015 Sep 23;3:58. doi: 10.1186/s40478-015-0237-8 (PMC4580349; doi:10.1186/s40478-015-0237-8)
Supplement: Additional file 4: — BIN1-Tau interaction is mediated by the Tau sequence from aa 212 to aa 231. Overlaid detail of 2D [1H, 15N] HSQC spectra of 100 μM 15N Tau-F5 [165–245] free in solution (gray) or with a 1.2 molar amount of GST-BIN1/SH3 (red, superimposed). Blue arrows link the corresponding resonances in the free (gray) and bound (red) states. (PDF 74 kb) [file 40478_2015_237_MOESM2_ESM.pdf]

Antibodies used and their respective dilutions.

Antibody	epitope	Western Blot dilution	Immunofluorescence Dilution	Reference
AT270	pT181	1/1,000		Pierce antibodies MN1050
pS199	pS199	1/1,000		LifeTechnologies 44-734G
Tau-1	pS199-S208			Millipore MAB3420
CP13	pS202	1/1,000	1/200	Gift of Peter Davies
AT8	pS202/pT205	1/1,000		Pierce antibodies MN1020
AT180	pT231	1/1,000		Pierce antibodies MN1040
RZ3	pT231	1/1,000	1/200	Gift of Peter Davies
PHF-1	pS396/pS404	1/1,000	1/200	Gift of Peter Davies
Tau	Total tau		1/400	Dako Cytomation A0024
Tau-5		1/1,000		LifeTechnologies AHB0042
BIN1 99D	MycBD	1/1,000	1/200	Millipore 05-449
BIN1 Anti SH3	SH3	1/1,000	1/200	Abcam ab27796
α-tubulin		1/1,000		Abcam ab24622
PSD95			1/500	SYSY 124012
Synaptophysin			1/500	SYSY 101004
Clathrin			1/400	Santa-cruz sc-6579
