# Supplementary material for: Tau phosphorylation regulates the interaction between BIN1’s SH3 domain and Tau’s proline-rich domain
Source: Acta Neuropathol Commun. 2015 Sep 23;3:58. doi: 10.1186/s40478-015-0237-8 (PMC4580349; doi:10.1186/s40478-015-0237-8)
Supplement: Additional file 12: — Supplementary methods. (PDF 89 kb) [file 40478_2015_237_MOESM12_ESM.pdf]

Supplemental experimental procedures

Phosphorylation of the Tau protein

The CDK2/CycA3 protein was prepared as primarily described in (1) and modified in (2). Phosphorylation of Tau by the CDK/CycA3 kinase is described in (2). The ERK kinase was prepared as described in (3). 100µM of ^{15}N-labelled recombinant Tau[165-245] was phosphorylated by incubation with 1 µM of activated ERK at 37°C during 3h in 200µL of phosphorylation buffer 50mM Hepes pH 8.0, 50 mM NaCl, 12.5 mM $MgCl_2$, 2.5mM ATP, 2mM DTT, 1 mM EDTA, 2mM EGTA and protease inhibitor cocktail (ROCHE, Complete Inhibitors without EDTA).

Rat brain extract was prepared by homogenizing rat brains (about 2g in 5 ml) in homogenizing buffer (10mM Tris.Cl pH 7.4, 5mM EGTA , 2mM DTT, 1µM okadaic acid (Sigma) supplemented with protease inhibitor cocktail. Ultra-centrifugation was next performed at 100,000g for 1 hour. The phosphorylation reaction of 10 µM ^{15}N-Tau protein (4) was performed at 37°C for 24h with 500 µl of rat brain extract in 10 mL of phosphorylation buffer (2 mM ATP, 40mM Hepes.KOH pH 7.3, 2mM MgCl2, 5mM EGTA, 2mM DTT complemented with a protease inhibitor cocktail and 1 µM okadaic acid (Sigma).

Enzymatic incubations were terminated by heating at 75°C fro 15 min and followed by centrifugation. The phosphorylation mixture was buffer-exchanged using desalting centrifugal devices (0.5ml bed of G25 resin, cut-off of 7KDa, Thermo Scientific Zeba Desalting Columns) against NMR buffer.

Co-localization measurement

Neurons were imaged with a Zeiss LSM 710 confocal imaging system using a 40x or 63x oil-immersion objectives. Z-stacks were 0.36 µm per step with a 1 AU pinhole. The co-localization coefficients were measured for each stack using ZEN 2012 software following the method already published by Manders et al (5).

Supplemental References

1. Welburn J, Endicott J (2005) Methods for preparation of proteins and protein complexes that regulate the eukaryotic cell cycle for structural studies. *Methods Mol Biol* 296:219–35.

2. Amniai L, et al. (2009) Alzheimer disease specific phosphoepitopes of Tau interfere with assembly of tubulin but not binding to microtubules. *FASEB J* 23(4):1146–1152.

3. Prabakaran S, et al. (2011) Comparative analysis of Erk phosphorylation suggests a mixed strategy for measuring phospho-form distributions. *Mol Syst Biol* 7:482.

4. Goedert M, et al. (1993) The abnormal phosphorylation of tau protein at Ser-202 in Alzheimer disease recapitulates phosphorylation during development. *Proc Natl Acad Sci U S A* 90(11):5066–70.

5. Manders EMM, Verbeek FJ, Aten JA (1993) Measurement of co-localization of objects in dual-colour confocal images. *J Microsc* 169(3):375–382.
